# Plasma-Based Degradation of Mycotoxins Produced by *Fusarium*, *Aspergillus* and *Alternaria* Species

**DOI:** 10.3390/toxins9030097

**Published:** 2017-03-10

**Authors:** Lars ten Bosch, Katharina Pfohl, Georg Avramidis, Stephan Wieneke, Wolfgang Viöl, Petr Karlovsky

**Affiliations:** 1 University of Applied Sciences and Arts, Faculty N, Von-Ossietzky-Strasse 99/100, 37085 Göttingen, Germany; stephan.wieneke@hawk-hhg.de (S.W.); wolfgang.vioel@hawk-hhg.de (W.V.); 2 Molecular Phytopathology and Mycotoxin Research, Georg-August-University Göttingen, Grisebachstrasse 6, 37077 Göttingen, Germany; pkarlov@gwdg.de; 3 Fraunhofer IST Application Centre, Von-Ossietzky-Strasse 100, 37085 Göttingen, Germany

**Keywords:** DBD, atmospheric pressure, low temperature plasma, mycotoxins, degradation

## Abstract

The efficacy of cold atmospheric pressure plasma (CAPP) with ambient air as working gas for the degradation of selected mycotoxins was studied. Deoxynivalenol, zearalenone, enniatins, fumonisin B1, and T2 toxin produced by *Fusarium* spp., sterigmatocystin produced by *Aspergillus* spp. and AAL toxin produced by *Alternaria alternata* were used. The kinetics of the decay of mycotoxins exposed to plasma discharge was monitored. All pure mycotoxins exposed to CAPP were degraded almost completely within 60 s. Degradation rates varied with mycotoxin structure: fumonisin B1 and structurally related AAL toxin were degraded most rapidly while sterigmatocystin exhibited the highest resistance to degradation. As compared to pure compounds, the degradation rates of mycotoxins embedded in extracts of fungal cultures on rice were reduced to a varying extent. Our results show that CAPP efficiently degrades pure mycotoxins, the degradation rates vary with mycotoxin structure, and the presence of matrix slows down yet does not prevent the degradation. CAPP appears promising for the decontamination of food commodities with mycotoxins confined to or enriched on surfaces such as cereal grains.

## 1. Introduction

Phytopathogenic fungi infect crops in the field (pre-harvest spoilage) while spoilage fungi colonize harvested commodities during storage (post-harvest spoilage). Besides the reduction of yield and quality, infection with fungal pathogens often leads to contamination with mycotoxins [1,2]. These toxic fungal metabolites have the potential to harm the health of consumers and livestock. Reduction of mycotoxin content in food and feedstuff is therefore an important goal of food and feed safety improvement.

Prevention of fungal contamination is the primary means of agricultural and food industry when it comes to compliance with maximum limits for mycotoxin content. In current production systems, however, even the best agricultural and manufacturing practices cannot fully prevent mycotoxin contamination. Degradation of toxic metabolites may be used to decontaminate food and feed products. Since most mycotoxins exhibit a high chemical stability, development of decontamination methods compatible with food quality standards is a challenging task. Over the last decades chemical, biological and physical strategies for the degradation of mycotoxins and the effect of food processing technologies on mycotoxin content were investigated extensively [3,4,5]. Among physical treatments mainly heating, irradiation and washing were studied. Mineral and organic mycotoxin binders are established since decades in animal production. More recently chemical and biological decontamination methods were studied. Among chemical methods, successful application of acids, bases, oxidizing agents, chlorinating agents, formaldehyde and ammoniation was described, especially for the decontamination of aflatoxin- and ochratoxin A-contaminated feeds. Although biological and enzymatic strategies have been developed since 1960’s [4] physical techniques still offer the most efficient removal of mycotoxins from food and feed [5].

Technical plasma is a novel physical method with a great potential as a post-harvest treatment method for mycotoxin mitigation. Plasma has been successfully used for sterilization and in plasma medicine [6,7,8,9,10,11]. Recent application of cold atmospheric pressure plasma (CAPP) in breaking seed dormancy and destruction of plant pathogens showed that the technology is suitable for sensitive biological materials [12,13,14,15]. Plasma of different types were used in studies of the inhibition of mycotoxin production and mycotoxin degradation. Ouf et al. [16] demonstrated inhibition of the synthesis of fumonisin B2 and ochratoxin A by *A. niger* after treatment with an atmospheric pressure argon plasma jet. Park et al. [17] successfully degraded aflatoxin B1 (AFB1), deoxynivalenol (DON) and nivalenol (NIV) within 5 s using a microwave-induced argon jet at atmospheric pressure.

Physical and chemical treatment of plant products bear a risk of reducing nutritional value and negatively affecting the palatability and sensory quality of the product. Long treatment duration, required by some methods for satisfactory decontamination of large quantities of goods, may increase the risk of these side effects. The effect of physical and chemical decontamination on nutritional value and quality of food commodities has rarely been systematically investigated. Because the energy of free electrons and excited ions and molecular species in CAPP exceeds the dissociation energy of a C-C bond, organic molecules in the discharge are subjected to unspecific degradation. This feature of CAPP was used to degrade chemically stable pollutants in gaseous phase (e.g., [18]). Low penetration depth of CAPP protects nutrients in bulk material from degradation, limiting degradation to a thin surface layer [19]. Kříž et al. [20,21] showed that the content of proteins and fibers as well as residual dry matter, nitrogen-free extract, fat and ash in intact barley grains were not significantly affected by treatment with CAPP while the content of selected mycotoxins was reduced by 20%–70%. We hypothesize that the confinement of mycotoxin contamination to the surface of grains accounted for the selectivity of the degradation in this study.

In the presented study, a dielectric barrier discharge (DBD) operated with ambient air at atmospheric pressure was utilized for the degradation of mycotoxins. Preliminary experiments using a similar setup and comparable dissipated discharge power applied on pea seeds (*Pisum sativum*) revealed no decline of seed germination rate, thus indicating negligible thermal effect due to plasma treatment [22]. The aim of this work was to test the effectiveness of cold atmospheric pressure air plasma based on a dielectric barrier discharge for the degradation of selected mycotoxins important in food and feed safety.

## 2. Results

### 2.1. Treatment of Cover Glasses

**Figure 1:**
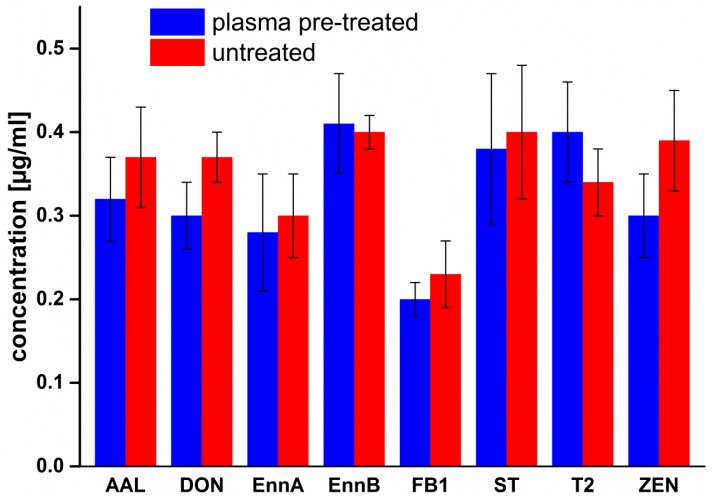
Effect of cover glass pretreatment on mycotoxins. Round cover-glasses were pre-treated with air-plasma for 5 s or not treated (controls). The further sample preparation was executed as described in Section 4.3. Significance of differences between treatments and control was tested by *t*-test at *p* = 0.05 with correction for multiple testing, according to Bonferroni. No significant difference was found.

Mycotoxin solutions were applied on untreated and 5 s pre-treated cover glasses in order to find out whether the activated surface of plasma-pretreated cover glasses (Section 4.3) might chemically affect mycotoxins. Mycotoxin content was determined as described in Section 4.4. As shown in Figure 1, pre-treatment of cover glasses with air plasma did not significantly affect mycotoxins on glass surface.

### 2.2. Treatment of Pure Mycotoxins and Fungal Extracts

**Figure 2:**
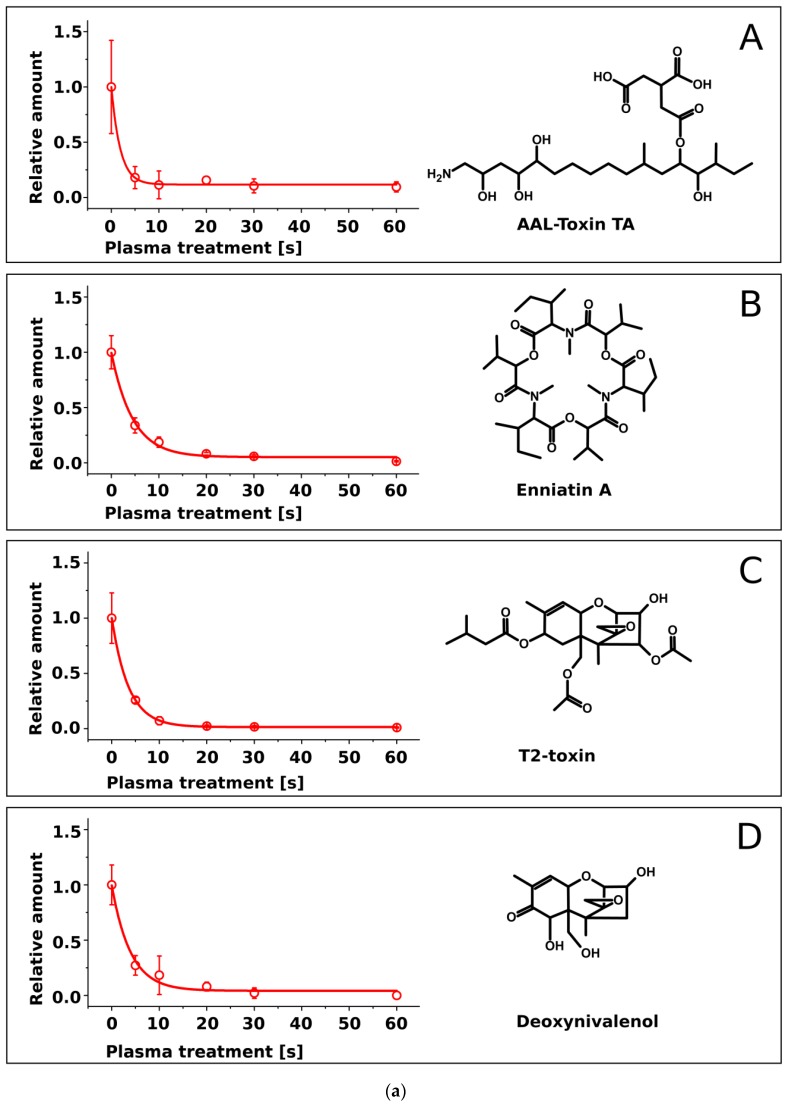

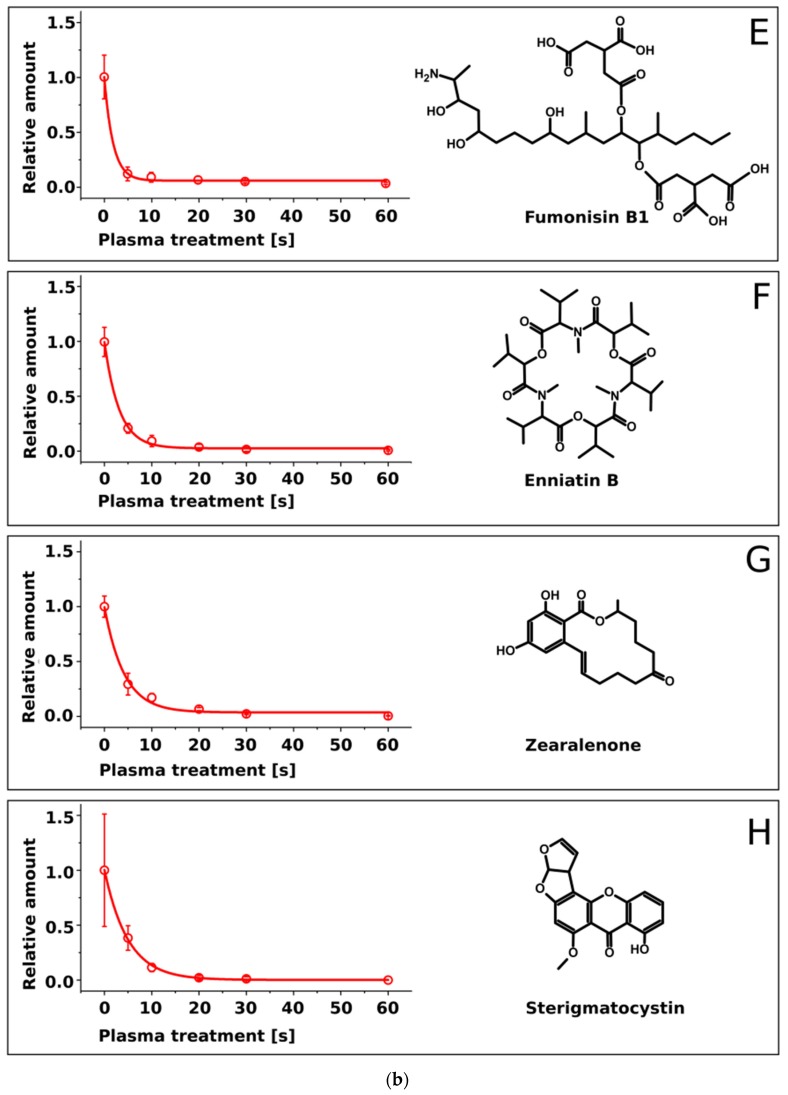
(**a**). Time-dependent decay of four pure mycotoxins exposed to air plasma (*N* = 5). (A) AAl-Toxin TA, (B) Enniatin A, (C) T2-toxin, (D) Deoxynivalenol; (**b**). Time-dependent decay of four pure mycotoxins exposed to air plasma (*N* = 5). (E) Fumonisin B1, (F) Enniatin B, (G) Zearalenone, (H) Sterigmatocystin.

AAL toxin, FB1, DON, ZEN, EnnA, EnnB, T2-toxin and ST as pure compounds covering the surface of cover glasses were subjected to air-plasma for 5 s, 10 s, 20 s, 30 s, and 60 s. HPLC-MS/MS analysis of residues on the glass surface revealed that the plasma treatment led to time-dependent degradation of all mycotoxins (Figure 2a,b). Searching for degradation products by HPLC-MS in a full-scan mode failed to detect any distinct MS signal.

The degradation kinetics seemingly followed an exponential decay. Since distinct differences among the toxins were apparent in their degradation rates, the measured data sets were fitted by an exponential function (Figure 2a,b).

(1) $$C(t)=y_{0}+C_{0}\cdot e^{-t/\tau }$$

C(t) is the concentration at time *t*, *C*_0_ is the initial concentration, *y*_0_ represents the threshold and τ represents the half-life. Half-life values calculated for all toxins by fitting Equation (1) to the data sets (Figure 2) are shown in Table 1.

**Figure 3:**
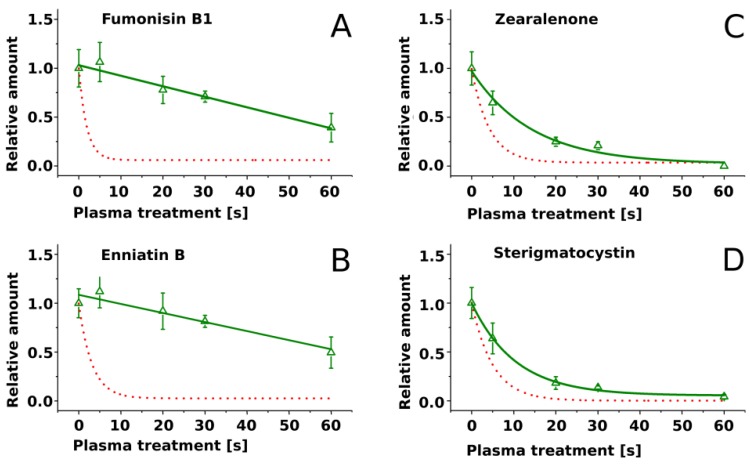
Time-dependent decay of mycotoxins embedded in rice extract (green line) exposed to air plasma (*N* = 5). (**A**) Fumonisin B1; (**B**) Enniatin B; (**C**) Zearalenone; (**D**) Sterigmatocystin. The red dotted line displays the decay slopes of the respective pure mycotoxin standards as shown in Figure 2b.

Four mycotoxins (FB1, EnnB, ST and ZEN) were selected to investigate the effect of matrix on mycotoxin degradation by plasma. Extracts of rice cultures of fungal strains producing these mycotoxins (*Fusarium verticillioides*, *Fusarium avenaceum*, *Aspergillus nidulans* and *Fusarium graminearum*), containing approx. 100 µg/mL of each toxin, were exposed to air plasma under the same conditions as pure compounds. The degradation rates were reduced as compared to pure compounds for all four mycotoxins (Figure 3). Particularly strong reduction of degradation rates was observed for FB1 and EnnB; nearly half of these toxins remained intact at the end of the treatment. Moreover, the course of the degradation of FB1 and EnnB was linear, contrasting to exponential decay of pure compounds. Degradation rates of ZEN and ST were reduced to a lesser extent and the progress of degradation of these toxins in matrix followed exponential decay similarly as the degradation of pure compounds (Figure 3).

## 3. Discussion

All investigated mycotoxins, when present as pure compounds, showed a distinct decay within a few seconds of plasma treatment duration and were reduced by approx. 2 log-ranges within 30 s as shown in Figure 2a,b. It is assumed that the energy dissipated in the discharge gap induces a combination of different degrading mechanisms acting on the toxins such as chemical reactions with reactive species generated in the plasma volume such as O, O_3_, OH, and NO_x_ [23,24] and/or decomposition after collision with electrons and ions [25,26] leading to cleavage of molecular bonds. Further reactions with plasma species can result in fragmentation and generation of volatile compounds. Decomposition of organic compounds into volatile products such as CO, CO_2_, and H_2_O during exposition of various polymer materials to oxygen-containing plasmas is a known phenomenon that was reported by several authors (e.g., [27,28,29]).

No stable residues of toxin degradation could be detected with HPLC-MS. Rapid degradation of toxin fragments into volatile products can be expected in analogy with the results of the study of Doraj and Kushner [27] in which degradation of polypropylene was elucidated. We assume that mycotoxin fragments were rapidly converted into volatile compounds which were immediately removed by the gas stream in the discharge gap. Therefore, future work will be dedicated to mass spectrometric investigation of the plasma effluent.

Our results (Figure 2a,b) are in accordance with the degradation of aflatoxin B1, DON, and nivalenol by Park et al. [17] who used a microwave argon plasma jet as well as by Ouf et al. [16] who demonstrated the reduction of fumonisin B2 and ochratoxin A by an atmospheric pressure argon kHz-operated jet. It should be kept in mind that sample temperature measured after the treatment by different approaches differed significantly: 35 °C were estimated after 9 min of treatment by Ouf et al. [16] and 105 °C after 5 s of treatment by Park et al. [17,30]. Elevated temperatures may cause thermal degradation. Since gas temperature and substrate temperature in this study did not exceed 60 °C, no thermal degradation of mycotoxin is expected.

Decay curves of individual toxins showed distinctly different degradation kinetics (Figure 2a,b). These differences were quantified by exponential fitting (Table 1). FB1 and AAL-toxin were degraded at the highest rate (τ = 1.9 ± 0.3) and ST at the lowest rate (τ = 5.0 ± 0.4).

The decay rates did not correlate with molecular mass. For example, EnnA with a molecular mass of 681.9 Da showed a similar decay rate as ST with a mass of 324.3 Da. The degradation rate might however be affected by the chemical structure. FB1 and structurally related AAL-toxin with long aliphatic chains were degraded rapidly, while ST with a compact structure of condensed aromatic rings had the highest half life. Most other mycotoxins with intermediate decay rates possessed mixed structures of condensed rings and aliphatic chains.

A relationship between the degradation rate of chemical compounds and their molecular structure was described by Gröning et al. [31] investigating plasma-based decay of polymers. These researchers suggested a buffering effect of aromatic structures on the degradation by low-pressure air-plasma. Klarhöfer et al. [32] reported higher resistance of lignin (which contains aromatic structures) exposed to an air DBD compared to cellulose and ascribed this observations to a similar mechanism. Aromatic structures occurring in mycotoxins might therefore slow down plasma-induced degradation. Future investigations will focus on degradation pathways, especially considering wide-spread toxins lacking aromatic rings. It is desirable to verify the postulated structural effects on degradation and identify reactive species in the plasma accountable for the degradation. Degradation of chemical constituents of solid materials by cold plasma is confined to thin surface layers [19]. Mycotoxins produced by Fusarium species in small-grain cereals are often enriched in the outer layers of grains [33,34]; this circumstance may facilitate selective degradation of mycotoxins in grains by cold plasma with a small loss of nutrients.

Mycotoxins imbedded in extracts of fungal cultures were degraded with lower rates than pure compounds. Presumably, components of the extracts scavenged reactive molecular species in the plasma, shielding mycotoxins from degradation. Activated components of the extracts are expected to rapidly react with other compounds including mycotoxins. Because the presence of matrix significantly reduced the degradation rates of mycotoxins, secondary chemical reactions apparently did not compensate for the loss of reactive molecular species by scavenging. We hypothesize that the effect of matrix on the degradation of other mycotoxins by plasma will be similar. In spite of the protective effects of culture extracts, significant decay of mycotoxins in culture matrix occurred, suggesting that plasma-based methods are promising for mycotoxin degradation in thin surface layers even in the presence of complex matrices.

## 4. Experimental Setup and Materials

### 4.1. Mycotoxin Standards

Pure mycotoxin standards of AAL toxin (TA1 + TA2), enniatin A (Enn A), enniatin B (Enn B), fumonisin B1 (FB1), sterigmatocystin (ST), deoxynivalenol, T2-toxin and zearalenone (ZEN) were purchased from Sigma Aldrich (Munich, Germany). Stock solutions were prepared in LC-MS grade methanol (Th. Geyer GmbH, Renningen, Germany).

### 4.2. Fungal Strains, Rice Cultures and Mycotoxin-Containing Fungal Extracts

*Fusarium verticillioides* VP2 [35] was kindly provided by Francesca Cardinale (University of Turin, Turin, Italy). *F. avenaceum* DSM 21724 was purchased from Deutsche Sammlung von Mikroorganismen und Zellkulturen (DSMZ, Braunschweig, Germany). *F. graminearum* IFA 66 was kindly provided by Marc Lemmens (Institute of Biotechnology in Plant Production, Tulln, Austria) via Thomas Miedaner (University of Hohenheim, Stuttgart, Germany). *F. graminearum* Fg71 was kindly provided by Thomas Miedaner. *Aspergillus nidulans* RDIT2.3 was kindly provided by Marko Rohlfs (University of Göttingen, Goettingen, Germany).

Fungal spores of *Fusarium* strains were produced according to Bai [36] with modification (Becker et al. [37]). The spores were suspended in sterile tap water and stored at −60 °C. Rice cultures were prepared as described (Nutz et al. [38]). All mycotoxins except fumonisins were extracted from 5 g rice cultures with 40 mL acetonitrile overnight with shaking, while fumonisins were extracted with methanol. An aliquot of 1 mL was dried, redissolved in 1 mL of methanol/water (1:1) and the concentration of mycotoxins was determined by HPLC-MS (see below). Remaining supernatants were dried in vacuum and the residues were dissolved and adjusted to a concentration of 100 µg/mL of ST (*A. nidulans* RDIT2.3), FB1 (*Fusarium verticillioides* VP2), Enn B (*F. avenaceum* DSM 21724) and ZEN (*F. graminearum* Fg71 and IFA 66).

### 4.3. Sample Preparation

Round cover-glasses (thickness 100 µm, diameter 16 mm) were placed onto microscopy slides and pre-treated with air-plasma for 5 s to facilitate an even spread of the toxin solution on the surface by increased surface tension (see e.g., Gerhard et al. [39]). Subsequently, 1.5 µL mycotoxin solution (100 µg/mL in methanol) were centrally applied onto the cover glass; the solution spread over the entire glass area spontaneously. After solvent evaporation at room temperature, the specimen underwent a plasma treatment at constant input power and varying treatment durations (0 s, 5 s, 10 s, 20 s, 30 s and 60 s). Then, the cover glasses were immersed in 0.5 mL methanol for at least 24 h to dissolve residual mycotoxins for analysis. 5 replicates per mycotoxin and treatment were used.

### 4.4. Mycotoxin Analyses

High performance liquid chromatography was performed as described (Ratzinger et al. [40]) using C18 column (Kinetex, 50.0 mm × 2.1 mm, particle size 2.6 μm; Phenomenex, Aschaffenburg, Germany); for the quantification of enniatins, 10 µM sodium acetate were added to the mobile phase. The analytes were ionized by electrospray and analyzed either by tandem mass spectrometry for quantification of mycotoxins or in a full scan mode while searching for degradation products using an ion trap detector 500 MS (Varian, Darmstadt, Germany). For the identification of mycotoxins retention time, *m*/*z* of molecular ions and fragmentation spectra were used. DON and ZEN were detected in a negative mode while all other mycotoxins were analyzed in positive ionization mode. The *m*/*z* values for molecular ions and mass transitions used were 522 > 328 for AAL-toxin, 722 > 686 for FB1, 662 > 549 for Enn B, 704 for Enn A, 325 > 310 for ST, 489 > 387 for T2-toxin, 355 > 295 for DON and 317 > 175 and 317 > 273 for ZEN. Quantification was carried out based on a linear calibration curve constructed with pure external standards. The estimated limits of quantification were 3 ng/mL for FB1, 5 ng/mL for Enn A/B, ST, AAL-toxin and T2-toxin, 10 ng/mL for DON 1 ng/mL and for ZEN. For all mycotoxins analyzed in positive ionization mode full-scan search for degradation products was carried out as described by Ratzinger et al. [40] followed by pairwise comparison of signal intensities after peak alignment and normalization [41].

### 4.5. Plasma Device

**Figure 4:**
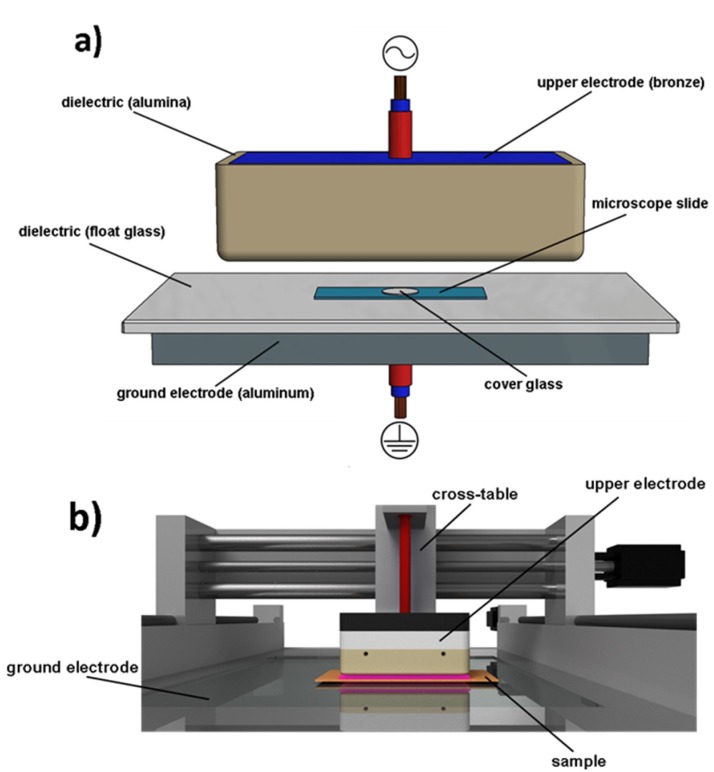
Principle of the electrode configuration (**a**) and scheme of experimental setup (**b**).

For plasma treatment, samples were positioned on a glass-insulated (float glass; thickness 4 mm) ground electrode (aluminum) so that the discharge gap between the samples and the upper electrode was 2 mm (Figure 4a). The upper electrode (145 × 85 × 28 mm, filling: bronze powder, dielectric: Al_2_O_3_, thickness of dielectric 3 mm) was connected to an alternating high-voltage (≈19 kV peak) pulse generator producing bipolar high-voltage pulses of a duration of approximately 1.9 µs and a repetition frequency of 17 kHz. 

Figure 4b depicts the plasma-setup and Table 2 the operational parameters. The upper high voltage electrode is attached to a cross-table and can be moved along 3 axes. One transition over the sample surface results in 5 s of plasma exposition at the chosen movement speed. The treatment of the samples was carried out by several consecutive sample transits by the upper electrode. During treatment compressed air flow was applied in order to cool the system and to homogenize the discharge. The gas temperature, estimation via optical emission spectroscopy (high resolution spectra applying an Echelle spectrograph, see e.g., [42,43]), and substrate temperatures, estimation via thermographic analysis (Fluke TiS Thermal Imager, Fluke Corporation, Everett, WA, USA), did not exceed 330 K or 60 °C after 60 s of treatment.

### 4.6. Measurement of Injected Power

The total energy converted in the gas discharge is an important parameter to characterize dielectric barrier discharges and was determined following the established cyclogram/Lissajous method [44]. The voltage/charge Lissajous figure (Q-U-plot) of the transferred charge and the applied voltage was used to calculate the energy dissipated into the system. The electrical parameters were measured using a high voltage probe (3 pF, 100 MΩ, Tektronix P6015A, Tektronix Inc., Beaverton, OR, USA) and a parallel circuit consisting of a capacitor (200 nF) and a resistor (1 kΩ). This parallel circuit was used for the charge measurement using a Yokokawa DL1740EL Dual 500 MHz oscilloscope (Yokogawa Electric Corp., Musashino, Tokyo, Japan) for digitalization. The voltage applied to the upper electrode was measured directly, whereas the transferred charge was determined by the measurement of the voltage drop on the capacitance (2 × WIMA FKP1, 100 nF). The calculated power injected to the system was approx. 500 W for all sample treatments. Considering the electrode geometry, a power density of ≈4/cm^2^ was calculated as depicted in Table 2.

## Figures and Tables

**Table 1:** Half-life at ≈4 W/cm^2^, molecular mass and chemical formula of the mycotoxins.

Mycotoxin	Half-Life *τ* [s]	Molecular Mass [Da]
Sterigmatocystin	5.0 ± 0.4	324.3
Enniatin A	4.5 ± 0.5	681.9
Zearalenone	4.2 ± 0.5	318.4
Deoxynivalenol	4.0 ± 0.7	296.3
T2-toxin	3.6 ± 0.1	466.5
Enniatin B	3.1 ± 0.2	639.8
AAL-toxin	1.9 ± 0.4	521.6
Fumonisin B1	1.9 ± 0.3	721.8

**Table 2:** Input parameters for experimental setup.

Input Parameter	Value
power density	≈4 W/cm^2^
discharge gap	2 mm
air flow	130 sl/min
appl. voltage	≈38 kV (p-p)
waveform	pulsed sine
gas temperature	T_rot_ ≈ 330 K
